# Spontaneous antral follicle formation and metaphase II oocyte from a non-stimulated prepubertal ovarian tissue xenotransplant

**DOI:** 10.1186/1477-7827-12-41

**Published:** 2014-05-15

**Authors:** Laura Lotz, Jana Liebenthron, Stephanie M Nichols-Burns, Markus Montag, Inge Hoffmann, Matthias W Beckmann, Hans van der Ven, Dagmar Töpfer, Ralf Dittrich

**Affiliations:** 1 Department of Obstetrics and Gynecology, Erlangen University Hospital, Friedrich-Alexander University Erlangen-Nürnberg, Erlangen, Germany; 2 Department of Gynecologic Endocrinology and Reproductive Medicine, University Women’s Hospital, Bonn, Germany; 3 ilabcomm GmbH, Eisenachstr. 34, 53757 St. Augustin, Germany; 4 Department of Reproductive Biology, University of Veterinary Medicine Hannover, Foundation, Buenteweg 2, 30559 Hannover, Germany

**Keywords:** Fertility preservation, Ovarian tissue cryopreservation, Prepubertal ovarian tissue, Xenotransplantation, Metaphase II oocyte

## Abstract

**Background:**

Current strategies in cancer treatment have markedly increased the rates of remission and survival for cancer patients, but are often associated with subsequent sterility. While there are various options available to an adult female depending on the patient’s particular situation, the only realistic option for preserving fertility in prepubertal females is to cryopreserve ovarian tissue. This is the first report of a morphologically mature oocyte collected from non-stimulated prepubertal ovarian tissue xenotransplants.

**Methods:**

Ovarian tissue from a 6 year old patient suffering from nephroblastoma was removed and cryopreserved for fertility preservation. The frozen-thawed ovarian tissue fragments were xenotransplanted to bilaterally oophorectomized severe combined immunodeficiency (SCID) mice to assess follicle development.

**Results:**

Antral follicle formation occurred post-xenotransplantation in a single ovarian fragment without exogenous hormone stimulation. A morphologically maturing oocyte was harvested from these follicles.

**Conclusions:**

Prepubertal human ovarian follicles and oocytes can be matured after xenotransplantation even without exogenous hormone stimulation. These results indicate that tissue collected from prepubertal patients can support fertility in cancer survivors.

## Background

Advances in the treatment of oncological diseases have led to greater survival rates for patients. However, treatment often includes gonadotoxic therapies, which can deplete germ cells and, in females, may result in premature ovarian failure in cancer survivors. While there are various routes available to an adult female depending on the patient’s particular situation, the only realistic option for preserving fertility in prepubertal females is to cryopreserve ovarian tissue [1-4]. This technique is a promising tool because the ovarian cortex contains a large pool of follicles [5,6] and provides a high developmental potential, especially in prepubertal patients [7,8].

The few transplantation studies of frozen-thawed prepubertal ovarian tissue that have been reported indicate that these grafts can support induction of puberty in young cancer survivors [9,10] and may presumably support reproduction at the appropriate time. These results, coupled with reports of more than 24 live births resulting from ovarian tissue cryopreservation and later transplantation to women [11], demonstrate the potential of the technique to move from the experimental realm to routine clinical practice.

We report here the occurrence of spontaneous antral follicle formation and oocyte maturation in an ovarian tissue transplant from a prepubescent girl (6 years old at the time of biopsy). To our knowledge, this is the first report of a morphologically mature oocyte collected from non-stimulated prepubertal ovarian tissue xenotransplants. These results further support the position that tissue collected from these young patients can support fertility in cancer survivors.

## Methods

### Patient

In 2009 ovarian tissue from a 6 year old patient suffering from nephroblastoma was removed and cryopreserved following informed consent and approval of the local university ethical committee (University Women’s Hospital, Bonn, Germany). About half of the ovarian cortex was removed via laparotomy in the therapeutically surgery for the nephroblastom from both ovaries. This patient and her parents were interested in cryopreserving ovarian tissue for fertility preservation and to contribute to research efforts in this area. Less than 5% of the frozen tissue was used for this experiment. A histological examination of the ovarian cortex was performed prior to cryopreservation in order to secure a sufficient amount of primordial follicles and exclude involvement of the tissue by malignancy.

### Ovarian tissue freezing and thawing

Details of the cryopreservation and thawing protocols for ovarian cortex tissue have been previously described. In brief, ovarian tissue biopsies were cut into pieces measuring 1 × 2 × 1 mm and equilibrated in a freezing solution containing 1.5 mol/L dimethylsulfoxide (DMSO) in Leibovitz medium. The pieces of ovarian tissue were then frozen in standard cryopreservation containers (1.8 mL Nunc cryovials) using a slow-cooling protocol with a closed freezing system (Icecube 14S; Sylab) with autoseeding. Cryopreserved ovarian tissue fragments were transported in a liquid nitrogen dewar to the Erlangen University Hospital (Erlangen, Germany) for thawing and transplantation experiments. Rapid thawing took place in a warm water bath (37°C until thawing). Tissue fragments were exposed in a step-wise manner to decreasing sucrose concentrations to release it from the protective cryopreservation medium [12].

### Animals

Female SCID mice (6 weeks old, Harlan-Winkelmann, Borchen, Germany) were housed in a high efficiency particulate air-filtered positive pressure room. Cages (Techniplast, Milano, Italy) were filter-topped and animals had access to food (Altromin 1314, Altromin, Lage, Germany) and water ad libitum under 12 h light: 12 h dark conditions. Groups of 5 mice were housed in 1 cage. Mice underwent a 1 week acclimation period upon arrival at the facility. All procedures and tests were performed under a laminar flow hood in a positive pressure room. Approval for the study was obtained from the local ethical committee on animal experimentation (Erlangen University Hospital, Erlangen, Germany). Animals were maintained in accordance with Animal Care and Use Committee regulations.

### Transplantation procedure

Surgery was performed under anesthesia with isoflurane (Isoflo® ad us. vet., Abott AG, Baar, Germany). Transplantation was performed irrespective of the estrous cycle stage. Mice ovaries were removed and 1 frozen/thawed human ovarian tissue fragment was placed in an intramuscular pocket of the neck. Grafting experiments were carried out in multiple rounds to ensure optimal technical handling and minimize the number of sacrificed animals.

### Treatment of the xenografted ovarian tissue

In this particular study, SCID mice and their grafted tissue were not exposed to any exogenous hormones. The finding was part of a larger study comparing prepubertal ovarian tissue growth *in vivo* versus *in vitro*. Exogenous hormone administration was not part of the experimental protocol in either case.

### Graft observations and oocyte retrieval

Study animals were observed daily for behavior, health and follicle growth was monitored with palpation of the neck. On the occasion that a lump was witnessed at the graft site, the animal was put under general anesthesia (isoflurane, 5%) and any apparent antral follicles were aspirated using a customized sterile needle (20G Supra, Karl Lettenbauer GmbH, Erlangen, Germany), specially beveled and sharpened for ease of aspiration and to minimize trauma to surrounding tissue at the site of action. The aspirate was inspected for the presence of oocytes. Products were placed into warmed and equilibrated culture medium without exogenous gonadotropins (Universal IVF Medium; Origio, Malov, Denmark), examined for meiotic stage and then placed into a 37°C incubator (5% CO_2_ in air) for *in vitro* culture. The animal was returned to its cage for continued observation following the procedure.

## Results

**Figure 1:**
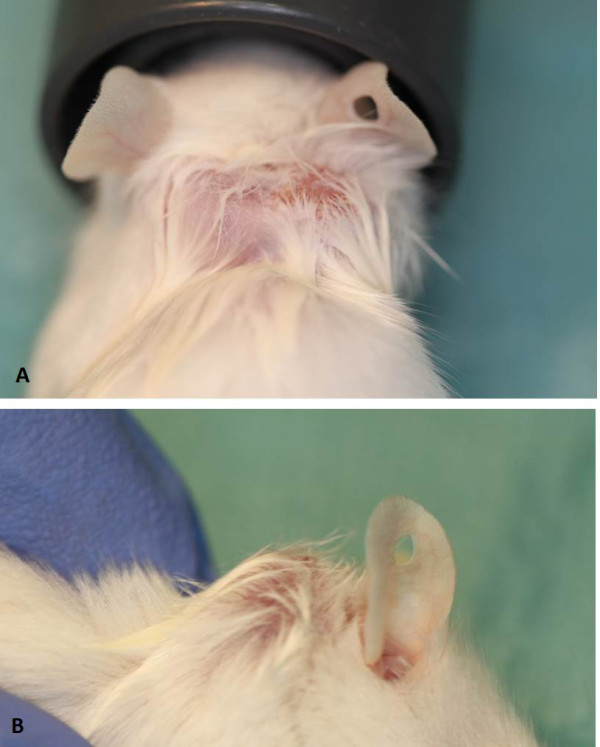
**Macroscopic view of large lumps seen at the site of the grafted prepubertal ovarian tissue Day 122 post-transplantation.** Antral follicles estimated to be ~7 mm in diameter. **(A)** Dorsal view; **(B)** Side view.

**Figure 2:**
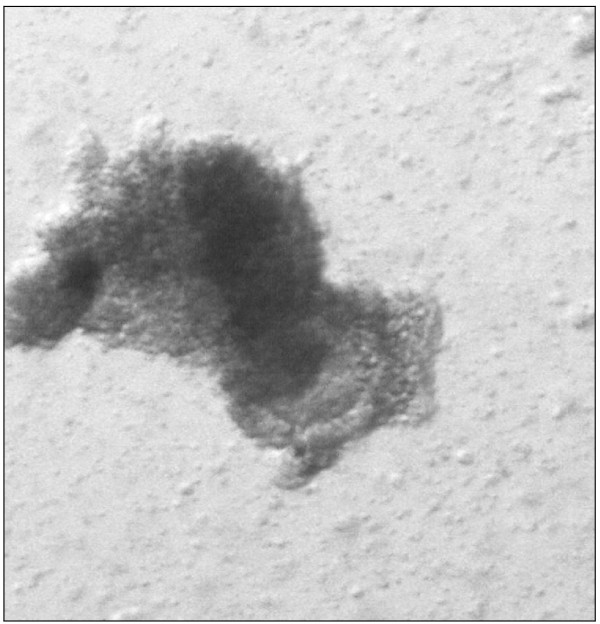
One maturing oocyte was recovered from the aspirate of an antral follicle from the xenotransplanted prepubertal ovarian tissue (32x magnification).

**Figure 3:**
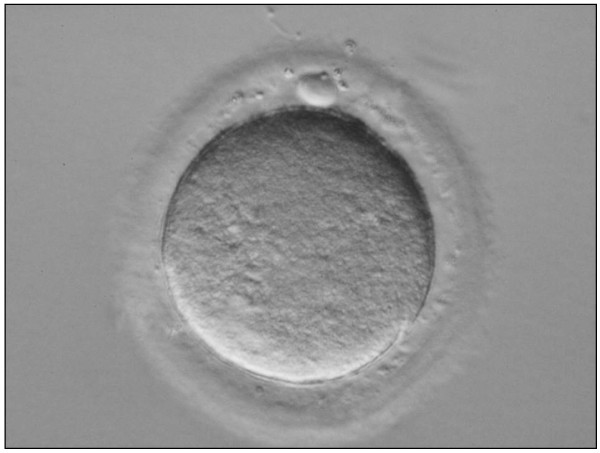
**Morphologically mature metaphase II oocyte following 22 h in *in vitro *culture.** Oocyte measured 150 μm in diameter (32x magnification).

On Day 122 post-transplantation, 2 large lumps, approximately 7 mm each in width, were noticed at the site of the grafted prepubertal tissue (Figure 1). One oocyte surrounded by a tight layer of cumulus and a sheet of granulosa cells was recovered from an antral follicle of the grafted tissue (Figure 2). This oocyte (diameter 150 μm) matured to the MII stage following 22 h of *in vitro* culture (Figure 3). The zona pellucida (ZP) was 14,6 μm.

## Discussion

Ovarian tissue cryopreservation and transplantation is proving to be a promising avenue for fertility preservation. Initial studies on the potential of this technique focused mainly on adult females for whom maintenance of fertility may be more of an immediate issue after cancer treatment. Information gleaned from these studies may be extrapolated to the adolescent female, for whom many of the current options of preserving fertility are not possible or appropriate. Optimization of ovarian tissue collection, preservation and later thawing and transplantation are of the utmost importance in either situation. In the adolescent female in particular, following full recovery from cancer, the goal of ovarian transplantation is to support the induction of puberty, establish hormonal cyclicity and the conditions for the possibility of an eventual pregnancy.

The prepubertal ovary has been demonstrated to contain a population of follicles in all stages of growth in exception of preovulatory stages [5,6]. However, there appear to be intrinsic differences between the adolescent and sexually mature ovary that prevent the adolescent ovary from developing fully mature follicles and oocytes *in situ*. It is not clear what these differences may be or what factors may be involved. Anderson et al. report that intrafollicular dynamics, local environment and maturational processes that change within the ovary as a girl ages are all likely involved to varying degrees [13]. Luyckx et al. [8] were the first to demonstrate follicles contained in prepubertal ovarian tissue can be expected to function, once transplanted, in a manner comparable to ovarian tissue from an adult or sexually mature female. In an effort to improve transplant outcome, the addition of exogenous hormone stimulation has been implemented [8,14]. Luyckx et al. [8] demonstrated success in survival and development of frozen-thawed preantral follicles from prepubescent patients after xenotransplantation in SCID mice and exogenous hormone stimulation. The result of finding a Met II oocyte in this study affirms the results published by Luyckx et al. [8]. The few studies that have reported results from the autotransplantation of frozen-thawed prepubertal ovarian tissue demonstrate the possibility of this tissue to induce puberty and provide some hope for other prepubertal patients [9,10]. Establishment of follicular development and hormonal activity in frozen-thawed transplanted prepubertal ovarian tissue has also been demonstrated in animal models (mouse: [15-17]; nonhuman primate: [18]; sheep: [19]). These results demonstrate the potential of prepubertal ovarian tissue and further success can be expected as our understanding of underlying processes and technique development evolve.

In the current study spontaneous antral follicle formation and oocyte maturation in a prepubertal ovarian tissue xenotransplant occurred which is in accordance to the finding that within grafts of adult women spontaneous follicle development can take place [20]. To our knowledge, this is the first report of antral follicular formation and oocyte maturation without exogenous hormone administration in a frozen-thawed ovarian tissue transplant obtained from a prepubertal patient. Follicle growth was estimated to be to approximately 7 mm in width and the obtained oocyte measured 150 μm. The zona pellucida (ZP) was thin (14,6 μm), but did not significantly vary from ZP witnessed in mature oocytes obtained from stimulated *in vitro* fertilization (IVF) cycles ([21]). This result complements reports on the potential of frozen-thawed prepubertal tissue to successfully establish follicular activity [9,10] and indicates for the first time that prepubertal tissue can produce mature oocytes after xenotransplantation, even without gonadotropin stimulation. This may be advantageous for graft survival since it has been shown that prolonged gonadotropin stimulation may cause a loss of primordial follicles in xenografts [22-24] or in vitro [25].

There will be cases when transplantation will not be indicated due to presence of malignant cells within the transplant [26-28]. Xenotransplantation could be an option to obtain oocytes which can be used for achieving pregnancies of these patients. *In vitro* maturation (IVM) of oocytes provides an alternative and complementary technique. This can be accomplished in several ways to include: supplementary oocyte aspiration from ovarian tissue prior to ovarian tissue cryopreservation [29,30], these oocytes may be immediately cultured to maturity or cryopreserved; *in vitro* culture of the ovarian tissue post-thaw to produce antral follicle development and harvest mature oocytes from the excised tissue either for immediate use in IVF or for cryopreservation. Another promising option is an artificial ovary with isolated follicles as very recently described [31]. These avoid the potential risk of reintroducing malignant cells and increase opportunities for the patient’s future reproductive health and options.

## Conclusions

Our finding suggests that it may be possible to accomplish oocyte maturation during *in vitro* culture of prepubertal ovarian tissue, whether within isolated encapsulated follicles or within the ovarian tissue slice itself [7,8]. The SCID mouse essentially acts as a kind of optimal incubator and ovarian growth within this system may be mimicked *in vitro* once ideal conditions have been established. Future studies will continue to focus on optimizing conditions for in vitro growth of ovarian tissue in parallel with the goals of resumption of hormonal cyclicity, follicle growth and activity, and oocyte maturation (cytoplasmic and morphological) in transplanted tissue.

## Competing interests

The authors declare that they have no competing interests.

## Authors’ contributions

LL, JL, SMN and RD designed the study, analyzed the data, and wrote the manuscript. RD and IH carried out the xenotransplantation procedures. MWB, MM, HvdV, DT and RD supervised the study. All of the authors read and approved the final manuscript.
